# Simultaneous Preconcentration of Triazole Fungicide Residues Using In-Situ Coacervative Extraction Based on a Double-Solvent Supramolecular System Prior to High Performance Liquid Chromatographic Analysis

**DOI:** 10.3390/molecules27196273

**Published:** 2022-09-23

**Authors:** Rachaya Buppasang, Jaruwan Palasak, Rawikan Kachangoon, Kraingkrai Ponhong, Norio Teshima, Rodjana Burakham, Supalax Srijaranai, Jitlada Vichapong

**Affiliations:** 1 Creative Chemistry and Innovation Research Unit, Department of Chemistry and Center of Excellence for Innovation in Chemistry, Faculty of Science, Mahasarakham University, Maha Sarakham 44150, Thailand; 2 Multidisciplinary Research Unit of Pure and Applied Chemistry (MRUPAC), Department of Chemistry and Center of Excellent for Innovation in Chemistry, Faculty of Science, Mahasarakham University, Maha Sarakham 44150, Thailand; 3 Department of Applied Chemistry, Aichi Institute of Technology, 1247 Yachigusa, Yakusa-cho, Toyota 470-0392, Japan; 4 Materials Chemistry Research Center, Department of Chemistry and Center of Excellence for Innovation in Chemistry, Faculty of Science, Khon Kaen University, Khon Kaen 40002, Thailand

**Keywords:** in situ coacervative extraction, double-solvent supramolecular system, triazole fungicides, extraction, HPLC

## Abstract

An in situ coacervative extraction (IS-CAE) based on a double-solvent supramolecular system coupled to liquid–liquid microextraction is investigated for extraction and enrichment of triazole fungicides. The formation of a double-solvent supramolecular system was generated by in situ formation and used as an extraction solvent for the coacervative extraction method. No disperser solvent was required. This new double-solvent supramolecular system has a higher extraction ability than any of its components alone. The different factors that could affect the extraction capability were studied and optimized, including the type of double extractant and its volume, salt addition, vortex time, and centrifugation time. Under optimum extraction conditions, this method provides high enrichment factors (EFs) of 73–318 with low limits of detection (LODs) of 0.3–1 μg L^−1^ and limits of quantitation (LOQs) of 1–3 μg L^−1^. In addition, the proposed method was prosperously applied for the determination of triazole fungicides in water, fruit juice, and soy milk samples.

## 1. Introduction

The selection of a suitable sample preparation method is important because it has a significant effect on the method’s sensitivity, selectivity, accuracy, reproducibility, and reliability [1]. Due to complex interfering substances and the presence of analytes at an ultra-trace level in real samples, various sample pretreatment techniques are needed [2] for clean up and matrix removal and preconcentration of target analytes. Many sample preparation techniques require high consumption of hazardous organic solvents which often generate waste during the process and are time-consuming. To overcome these problems, miniaturized extraction techniques have been investigated. Nowadays, modern trends in sample preparation techniques are affected by the concepts of green and sustainable solvents, especially in liquid–liquid microextraction [3]. According to the requirements of the sustainable sample preparation process, green alternative solvents should have various characteristics, which include nontoxicity, low energy consumption, dissolution of a large spectrum of solutes, and fewer steps [4]. The requirement of environmentally friendly solvents is gradually improving. Consequently, it is important to design and to develop an environmentally friendly alternative solvent in sample preparation methods.

Recently, new classes of extraction solvents, namely supramolecular solvents (SUPRAS), have been investigated. They are nanostructured liquids that form automatically in colloidal suspensions of amphiphiles via the phenomena of self-assembly and coalescence [5]. Due to their unique properties, SUPRAS have been better substitutes for conventional organic solvents for sample preparation before chromatographic techniques [6]. Their physico-chemical properties, which make them very attractive as an alternative extraction solvent in microextraction techniques, include: (i) their ability to interact with analytes via several interactions such as ionic bonding, hydrogen bonding, π-cation, and hydrophobic interaction, leading to an improvement in extraction efficiency and (ii) tunability by altering either the type or concentration of amphiphiles [7]. In addition, SUPRAS are tunable solvents and the properties of the solvents can be easily changed by altering the group of the amphiphiles [8]. Moreover, they are environmentally friendly solvents produced from inexpensive amphiphiles, in which the coacervation occurs rapidly at room temperature [9], in which the pH, salt, and the solvent are also affecting the coacervation.

Triazole fungicides are a group of highly effective systemic fungicides that contain a hydroxyl group (ketone group), a substituted phenyl group, and a 1,2,4-triazole group in the main chain [10]. They have a wide fungicidal spectrum and good control effects on a variety of crop diseases. Owing to their antifungal properties, they are widely used for preventing and controlling diseases and are widely used in agriculture for control of various fungal diseases such as powdery mildew, gray mold, spotted deciduous disease, black star disease, brown spot disease, and rust disease, in agricultural products such as fruits, vegetables, legumes, and grain crops [11,12]. However, triazole fungicides have high stability and lipophilicity, long residual duration, and are not easily degraded, which leads to easy accumulation in human and environmental media [10]. In order to protect human health, the Codex Alimentarius Commission (CAC) has established standards/regulations for the maximum residue limits (MRLs) of triazole fungicides in different matrices. For example, the MRL of hexaconazole, triadimefon, and bitertanol is 0.01–0.02 mg kg^−1^; the MRL of tebuconazole is 0.02–5.0 mg kg^−1^; and the MRL of myclobutanil is 0.05–3.0 mg kg^−1^ [10]. Therefore, it is necessary to establish a fast and efficient method for analyzing triazole fungicides in agricultural products [13].

In this study, we developed an in situ coacervative extraction (IS-CAE) based on a double-solvent supramolecular system coupled to liquid–liquid microextraction for extraction and enrichment of triazole fungicides prior to high-performance liquid chromatographic analysis. The phase separation obtained after centrifugation was formed by mixing the double-solvent supramolecular system. No organic solvent or heating were required. The proposed coacervative extraction strategy is far greener and more sustainable than the currently employed coacervative extraction. The important parameters affecting the IS-CAE were optimized and the resulting method was also applied to water, fruit juice, and soy milk samples.

## 2. Results and Discussion

### 2.1. Optimization of In Situ Extraction (IS-CAE) Procedure

In order to obtain high extraction efficiency, different experimental factors that affect the efficiency of the in situ coacervative extraction (IS-CAE) procedure were investigated and optimized. The peak area of the studied triazoles was used for the evaluation based on the one variable-at-a-time method, and all experiments were performed in triplicate using standard solution at a concentration of 100 μg L^−1^ of each analyte.

**Figure 1:**
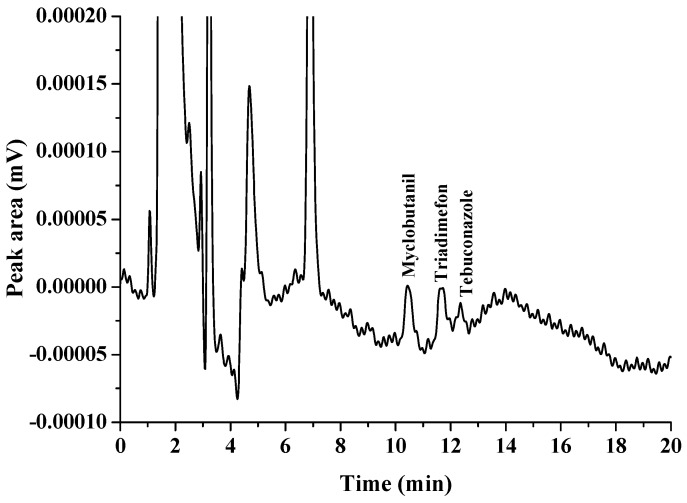
Extraction of triazole fungicides using 1-dodecanol as a solvent.

**Figure 2:**
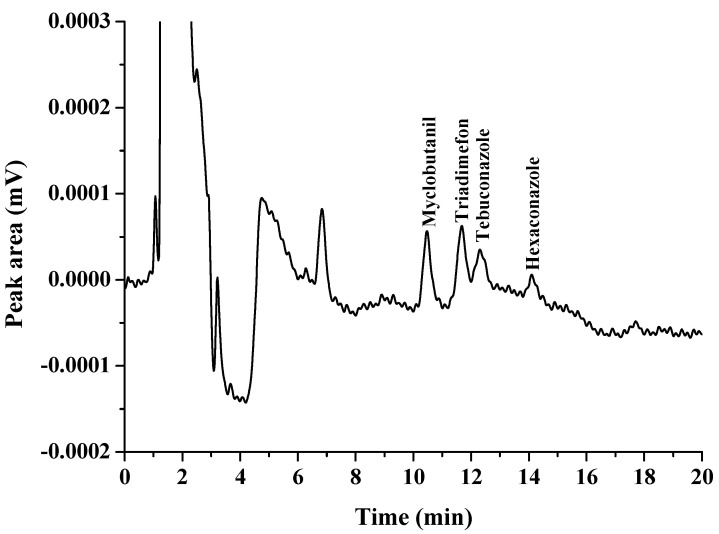
Extraction of triazole fungicides using 1-undecanol as a solvent.

**Figure 3:**
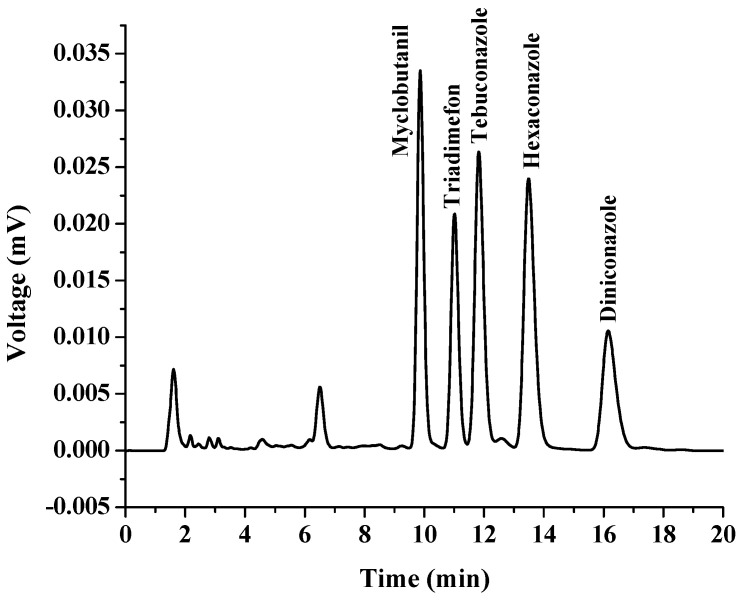
Extraction of triazole fungicides using mixture solvent (1-dodecanol and 1-undecanol).

**Figure 4:**
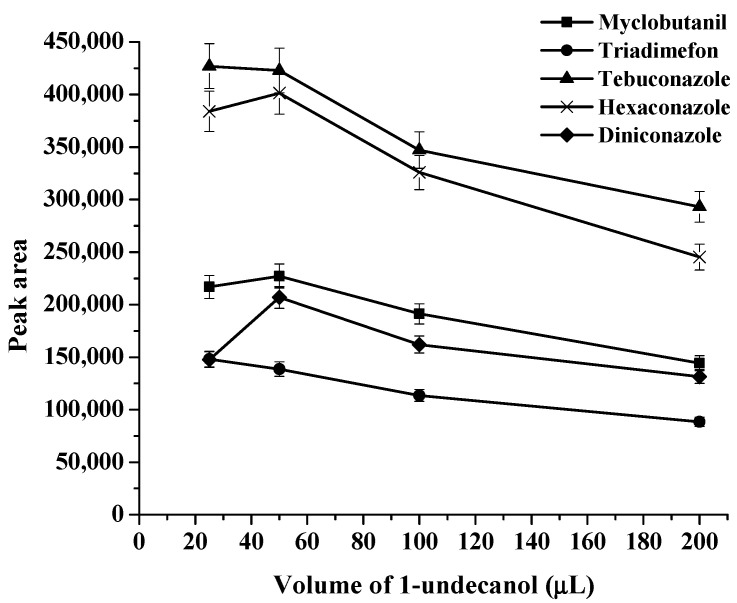
Effect of volume of 1-undecanol on extraction efficiency.

**Figure 5:**
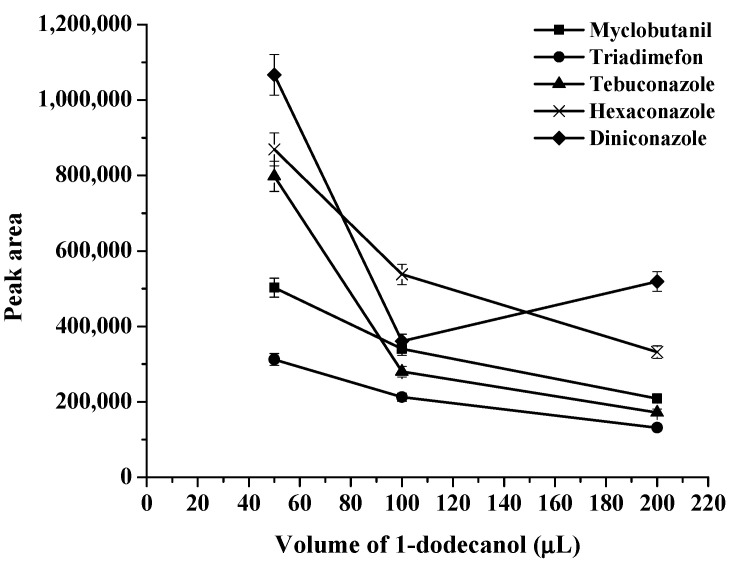
Effect of volume of 1-dodecanol on extraction efficiency.

The choice of a suitable double extraction solvent is important because this is a significant parameter in the proposed method. A double extraction solvent must have a melting point close to room temperature, high extraction efficiency, less toxicity, and low solubility in the aqueous phase [14]. Therefore, 1-dodecanol (melting point 24 °C), and 1-undecanol (melting point 24 °C) were selected as extraction solvents in this work. First, each of the solvents was studied as an extraction solvent, and the results were compared with their double mixture with a specific ratio (as shown in Figure 1, Figure 2 and Figure 3). It was found that the extraction efficiency of triazoles using the double mixture resulted in a higher extraction efficiency than the single solvent. Therefore, a double mixture solvent (1-dodecanol and 1-undecanol) was used for further study. The formation of the double-solvent supramolecular system was generated by in situ formation. Therefore, the 1-undecanol and 1-dodecanol volumes were studied. In this work, the 1-undecanol volume was studied in the range of 25–200 μL (as shown in Figure 4). The results showed that a high extraction efficiency in terms of peak area was obtained with 50 μL of 1-undecanol. The volume of 1-dodecanol was investigated in the range of 25–200 μL (as shown in Figure 5). The results showed that with 25 μL of 1-dodecanol the phase did not occur. A high extraction efficiency in terms of peak area was obtained with 50 μL of 1-dodecanol. Therefore, the most suitable proportion of double extractant was selected to be 1:1 of 1-undecanol/1-dodecanol to achieve the best extraction efficiency in this method.

To evaluate the salt effect on the efficiency of the in situ extraction procedure, various tests were carried out using different concentrations of salt in the range of 0–10% (*w/v*) NaCl (data not shown). The results indicated that by increasing NaCl from 0 to 5% (*w/v*), the peak area of triazoles remained nearly constant. At higher percentages, the analytical signal of the analytes decreased due to the dilution effect. Therefore, the experiments were carried out in the absence of any salt.

**Figure 6:**
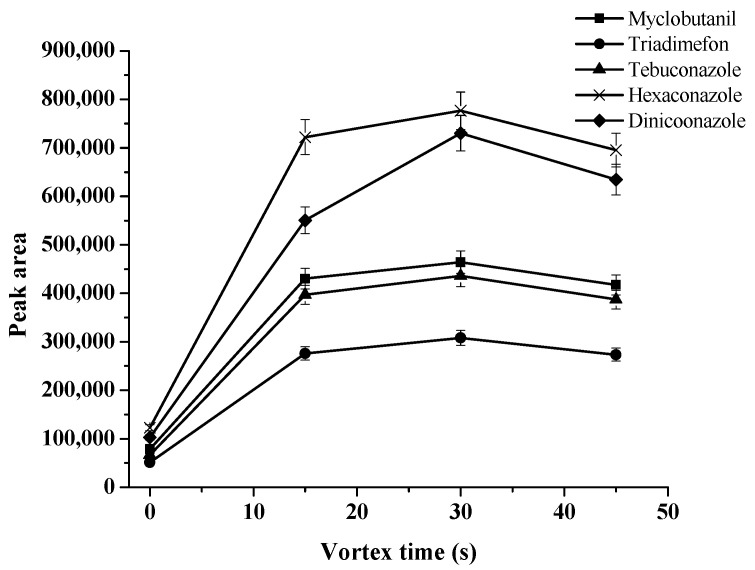
Effect of vortex time on extraction efficiency.

The vortex of the solution can accelerate the transfer of an analyte from an aqueous solution to the double-solvent supramolecular phase. An appropriate dispersion occurs in the presence of a strong vortex. Therefore, the vortex time was examined at 0, 15, 30, and 45 s. The results obtained (Figure 6) showed that the maximal analytical signals were observed at 30 s. Therefore, 30 s of vortex time was chosen for the next experiments.

**Figure 7:**
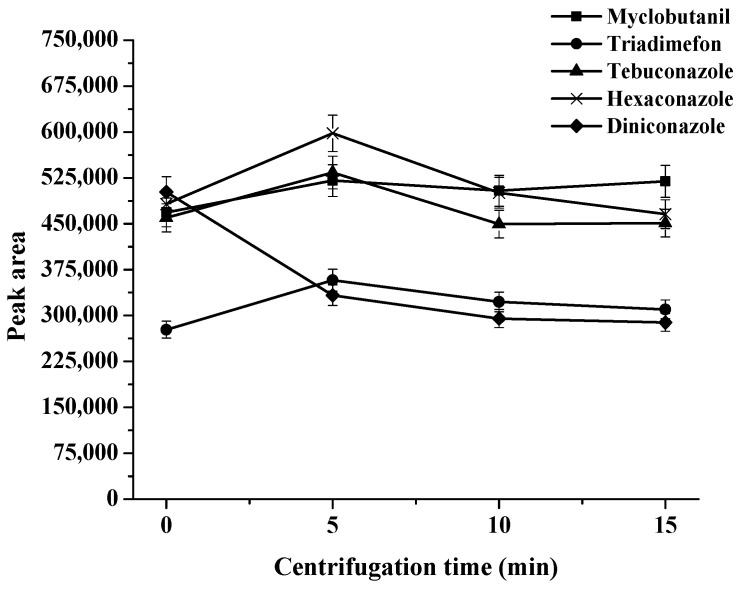
Effect of centrifugation time on extraction efficiency.

The centrifugation times were studied at 0, 5, 10, and 15 min at 2500 rpm. There were no significant differences in extraction efficiency found by increasing the centrifugation time from 5 to 15 min (as can be seen in Figure 7). Incomplete phase separation was obtained at 0 min (without centrifugation). In order to minimize the extraction time, therefore, 5 min was selected as the optimum centrifugation time.

### 2.2. Analytical Performance of the Proposed Extraction Method

**Figure 8:**
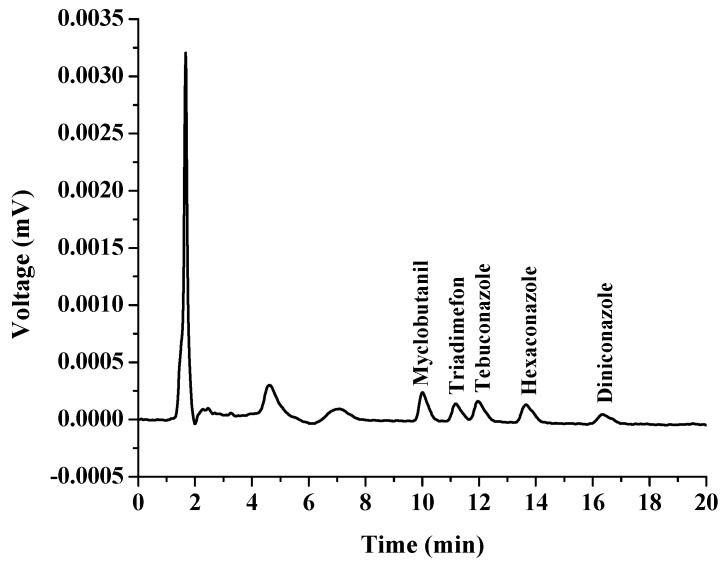
Chromatogram of standard triazole fungicides obtained by direct HPLC analysis. The concentration of each standard was 100 µg L^−1^.

**Figure 9:**
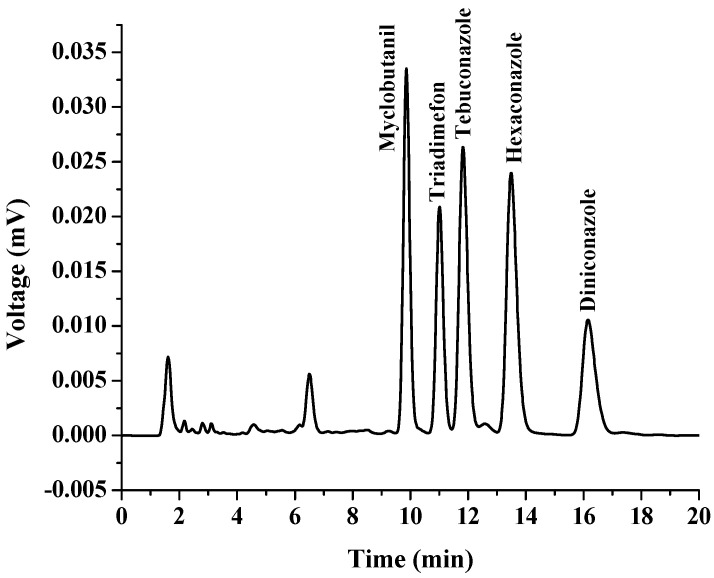
Chromatogram of standard triazole fungicides obtained with preconcentration using the proposed in situ coacervative extraction based on a double-solvent supramolecular system. The concentration of all standards was 100 µg L^−1^. Conditions: Sample 10 mL, double SUPRA (50 µL of 1-dodecanol and 50 μL of 1-undecanol), vortex time 30 s, and centrifugation 2500 rpm for 5 min. Finally, collection of the top layer for HPLC analysis.

Linear ranges (LR), coefficient of determination (R^2^), limit of detection (LOD), limit of quantification (LOQ), relative standard deviation (RSD) and enrichment factors (EFs) were calculated to validate the proposed method. All the data were obtained by conducting three replicates for each experimental test and the results are shown in Table 1. The calibration curve was constructed by plotting the peak area ratios against concentrations of triazoles. The linearity range was found to be from 0.3 to 1000.0 μg L^−1^, with a high coefficient of determination (R^2^ > 0.999), which showed an excellent level of linearity. The LODs and LOQs of the analytes were determined according to signal-to-noise ratios of 3 and 10, respectively. The results showed that the LODs ranged from 0.3 to 1.0 μg L^−1^, while the LOQs were within 1–3 μg L^−1^. The precision was studied by intra-day RSDs (*n* = 3) and inter-day RSDs (*n* = 3 × 3), which were lower than 4.84% and 4.95%, respectively. The EFs, were calculated using the ratio of the extracted analyte concentration in extraction phase to its initial concentration in aqueous sample solution, and were in the range of 73–318. The chromatograms of the triazoles obtained by direct HPLC and the proposed in situ coacervative extraction procedure are presented in Figure 8 and Figure 9, respectively.

### 2.3. Real Sample Analysis

The applicability of the proposed in situ coacervative extraction (IS-CAE) coupled to the HPLC method was investigated to determine triazole fungicide residues in water, fruit juice, and soy milk samples. To investigate the matrix effect of real samples, a matrix-match calibration procedure was carried out. A set of matrix-matched calibration curves was prepared by extracting representative water, fruit juice, and soy milk samples spiked with 3.0–1000.0 μg L^−1^ of each target analyte. The studied triazole fungicides exhibit wide calibration capability and good linearity, with R^2^ values greater than 0.99 for all studied samples.

The matrix effect (ME) was calculated by comparing the ratio of the slopes of the matrix-matched curve to that of the solvent (as shown in Equation (3)). Generally, An ME between 80–120% indicates no matrix effects, an ME between 50–80% or 120–150% refers to minor matrix effects, and an ME < 50% or >150% indicates major matrix effects [15,16]. As shown in Table 2, from no ME to a minor ME was observed for the water and fruit juice samples and major MEs were found in the soy milk samples.

**Figure 10:**
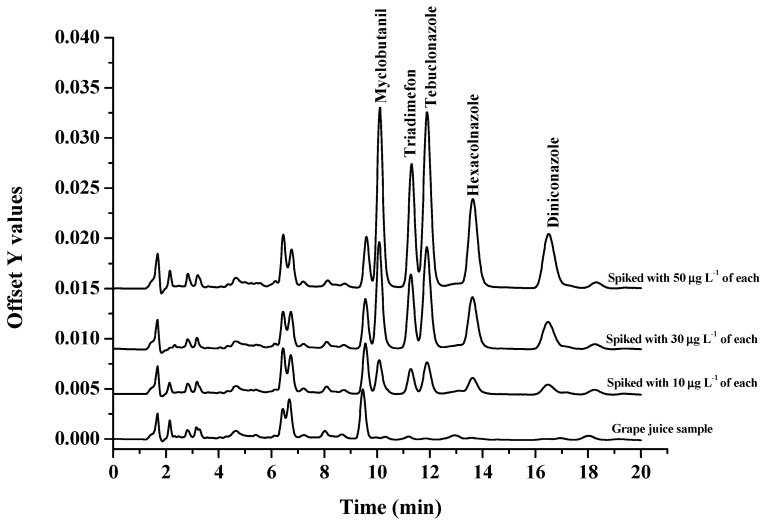
Chromatograms of the grape sample and spike grape samples at three concentration levels (10, 30, and 50 μg L^−1^ of each analyte).

The accuracy and repeatability of the in situ extraction coupled to the HPLC method were evaluated by spiking the real samples with five triazole fungicides at concentration levels of 10, 30, and 50 μg L^−1^. The results were shown in Table 3. Extraction recoveries in the range of 77–117% were obtained with RSDs in the range of 0.1–10.7%. Figure 10 illustrates the chromatograms of the blank and spiked (grape juice) samples. Based on these observations, it can be concluded that the proposed in situ extraction coupled to the HPLC method has excellent applicability for the selective extraction of five triazole fungicides in various samples.

### 2.4. Comparison of the Proposed in Situ Coacervative Extraction (IS-CAE) Method with Other Previous Extraction Methods

To highlight the outstanding points of the developed method, some major characteristics were compared with those that have been obtained from other reported methods [10,17,18,19,20], as listed in Table 3. As compared with other methods, the established method has various advantages, such as the use of a green extraction solvent, a short extraction time (6 min), and avoidance of the use of a disperser solvent. Moreover, the proposed method exhibits a favorable linear range, low LOD, acceptable recovery, and high enrichment factor. Therefore, the proposed method is fast, simple, and environmentally friendly.

## 3. Experimental Methods

### 3.1. Chemicals and Reagents

All chemicals and reagents used in this work were of analytical grade. Five triazole fungicides (myclobutanil (MCBT), triadimefon (TDF), tebuconazole (TBZ), hexaconazole (HCZ), and diniconazole (DCZ)) from Dr. Ehrenstorfer GmbH (Augsburg, Germany)were used. Methanol (Merck, Darmstadt, Germany) was used to prepare the stock solution of each fungicide (1000 mg L^−1^) and stored in refrigerator at 4 °C under light protection until analysis. HPLC-grade methanol and acetonitrile were obtained from Merck (Darmstadt, Germany). 1-Undecanol and 1-dodecanol were purchase from Sigma-Aldrich (Darmstadt, Germany). Deionized water with the resistivity of 18.2 MΩ.cm was obtained from a Type 1 Simplicity^®^ ultrapure water system (Merck, Darmstadt, Germany). All solutions were filtered through a 0.45 μm nylon membrane filter before injected into the HPLC system.

### 3.2. Instrumentations

The chromatographic analysis of triazole fungicides was performed on a Waters 1525 Binary HPLC pump (Water, MA, USA) equipped with a diode array detector (DAD). The stationary-phase column was a Purospher^®^ STAR RP-18 endcapped (4.6 × 150 mm^2^, 5 µm) column (Merck, Darmstadt, Germany) with the column temperature maintained at ambient temperature. The mobile phase consisted of acetonitrile and water, and the separation was carried out under an isocratic elution of 50:50 (%*v*/*v*), and the flow rate was 1.0 mL min^−1^, the injection volume was 20 μL, and the detection wavelength was set to 220 nm.

### 3.3. In-Situ Coacervative Extraction (IS-CAE) Procedure

**Figure 11:**
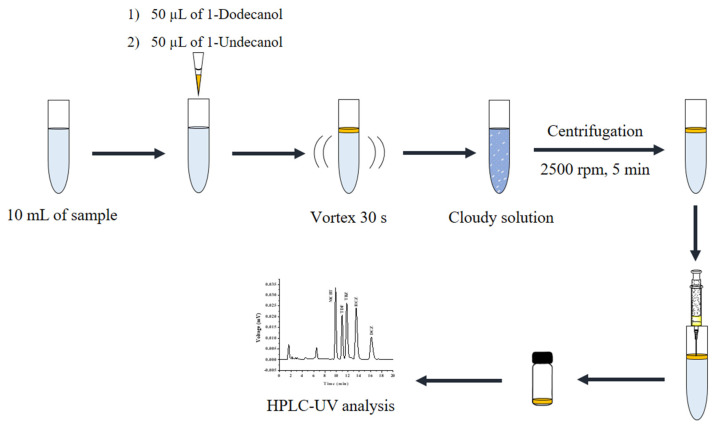
Schematic illustration of the proposed microextraction using in situ coacervative extraction based on a double-solvent supramolecular system for triazole fungicides and HPLC analysis. Conditions: Sample 10 mL, double SUPRA (50 μL of 1-dodecanol and 50 μL of 1-undecanol), vortex time 30 s, and centrifugation 2500 rpm for 5 min. Finally, collection of the top layer for HPLC analysis.

The standard solution of triazoles (or sample solution) of 10.00 mL was mixed with 50 μL of 1-dodecanol and 50 μL of 1-undecanol in the centrifuge tube. Then, the solution was vortexed for 30 s. After that, the emulsion was centrifuged at 2500 rpm for 5 min to complete the phase separation. The reconstituted solution was collected before injecting into the HPLC system. A schematic diagram of the proposed microextraction procedure is shown in Figure 11.

### 3.4. Sample Preparation

#### 3.4.1. Water Samples

The water samples were collected from different areas located near rice fields in Maha Sarakham province, northeastern of Thailand, and were filtered through a 0.45 μm nylon membrane filter (Millipore, Burlington, MA, USA) before extraction using the proposed method.

#### 3.4.2. Fruit Juice Samples

Commercial grape and apple juice samples, available in local supermarkets, were collected for analysis. Before analysis, a 30.0 mL aliquot of fruit juice was centrifuged at 3500 rpm for 15 min, and was filtered through a Whatman No. 42 filter paper. Then, the filtrate was filtered through a 0.45 μm nylon membrane filter before extraction using the proposed method.

#### 3.4.3. Soy Milk Samples

Commercial soy milk samples were purchased from a local supermarket in Kantarawichai Distinct, Maha Sarakham Province, Northeast, Thailand. Proteins and fats in 1 mL samples were precipitated by shaking vigorously with acetonitrile and trifluoroacetic acid (5:1, *v*/*v*). then, the mixture was vortexed (1500 rpm, 3 min) and centrifuged at 4500 rpm for 10 min. The supernatant was extracted by using the coacervative extraction procedure (see Section 2.3). For the fortification of samples, standards of triazole were spiked into milk samples prior to protein and fat separation.

### 3.5. Calculation of Enrichment Factor (EF), Relative Recovery (RR), and Matrix Effect (ME)

The EF is the ratio between the concentration of analyte in the sediment phase (C_sed_) and the initial concentration of analyte in the aqueous sample solution (C_0_). To study the effect of experimental conditions on the extraction efficiency, the EFs were calculated according to the following equations:

EF = C_sed_/C_0_ (1)

The %RR was defined as the %amount of analyte recovered from matrix (real samples) with reference to the extracted standard (standard spiked into the same matrix):

(2) $$\mathrm{RR}(\%)=\frac{C_{\mathrm{found}\;}{\;-\;C}_{\mathrm{real}}\;}{C_{\mathrm{added}}}\;\times \;100$$

where C_found_ is the concentration of analyte after adding a known amount of working standard to real sample, C_real_ is the analyte concentration in real sample, and C_added_ represents the concentration of a known amount of working standard that was spiked into the real samples.

ME (%) is expressed as the ratio of the slopes obtained from calibration curves of each analyte spiked into the samples to the slopes obtained after extraction using the proposed method, according to the following equation:

(3) $$\mathrm{ME}(\%)=\frac{\mathrm{slope}\;\mathrm{of}\;\mathrm{spiked}\;\mathrm{real}\;\mathrm{sample}\;}{\mathrm{slope}\;\mathrm{of}\;\mathrm{standard}\;\mathrm{solution}}\;\times \;100$$

## 4. Conclusions

In this study, an in situ coacervative extraction (IS-CAE) based on a double-solvent supramolecular system combined with HPLC was investigated for the analysis of triazole fungicides. The advantages of this method include a simple and inexpensive operational procedure, environmentally friendly, dispersive-solvent-free, and low organic solvent consumption. In this method, two long normal chain alcohols are in situ formed in the sample solution in which coacervative extraction was performed. This new supermolecule is used as an extractant system, which has a higher extraction power than any of its components alone. Therefore, IS-CAE fulfills the demand of green and sustainable analytical chemistry. In addition, this method was successfully applied to determine triazole fungicide residues in water, fruit juice, and soy milk samples, by providing satisfactory recoveries.

## Figures and Tables

**Table 1:** Analytical performances of the present method.

Analyte	Linear Range (μg L^−1^)	R^2^	LOD (μg L^−1^)	LOQ (μg L^−1^)	Intra-Day Precision (*n* = 3), RSD (%)		Inter-Day Precision (*n* = 3 × 3), RSD (%)		EF (C_ex_/C_o_)
					t_R_	Peak Area	t_R_	Peak Area	
Myclobutanil	3–1000	0.9999	1.0	3.0	1.89	2.50	1.96	3.42	74.82
Triadimefon	3–1000	0.9995	0.3	1.0	1.98	4.84	1.99	4.84	103.50
Tebuconazole	3–1000	0.9995	0.3	1.0	1.03	3.89	1.04	4.62	317.49
Hexaconazole	3–1000	0.9998	0.3	1.0	0.56	2.70	0.65	3.13	137.33
Diniconazole	3–1000	0.9995	1.0	3.0	0.61	3.52	0.75	4.95	73.81

R^2^: coefficient of determination; LOD: limit of detection; LOQ: limit of quantification; RSD: relative standard deviation; EF: enrichment factor; t_R_: retention time.

**Table 2:** Matrix effect (ME, %).

Sample	Myclobutanil	Triadimefon	Tebuconazole	Hexaconazole	Diniconazole
Water I	78.83	77.95	71.31	82.18	82.89
Water II	87.78	71.37	79.63	84.38	86.63
Grape juice	75.00	75.00	83.33	75.00	100.00
Soy milk I	71.12	49.94	155.59	73.45	155.53
Soy milk II	72.25	48.83	152.14	77.72	145.55
Soy milk III	75.15	49.98	145.54	78.83	147.72

**Table 3:** Comparisons of the proposed IS-CAE method with other methods for the determination of triazole fungicides.

Method	Analyte/ Sample	Linear Range	Limit of Detection (LOD)	%Recovery	Enrichment Factor (EF)	Reference
SVME	Triadimefon and triadimenol/beer samples	0.5–50 µg L^−1^ for triadimenol and 1.0–100 µg L^−1^ for triadimefon	0.24–0.99 µg L^−1^	84–100	-	[17]
ATPS	Triazole fungicides/vegetable samples	0.100–30 µg mL^−1^	0.03113–0.3525 µg mL^−1^	71.57–107.8	-	[18]
SBSE	Triazole fungicides/grape and cabbage samples	0.1–500 µg L^−1^	0.022–0.071 µg L^−1^	80.7–111	49–57	[10]
VA-DLLME	Triazole fungicide, herbicide, pesticide and insecticide/fruit juice samples	149–500,000 ng L^−1^	45–78 ng L^−1^	55–89	1382–2246	[19]
CD-DLLME	Triazole and strobilurin fungicides/ water, juice, and vinegar samples	1–100 µg L^−1^	0.3 µg L^−1^	83.0–103.2	124	[20]
IS-CAE	Triazole fungicides	3–1000 µg L^−1^	0.3–1.0 µg L^−1^	77–117	73–318	This work

SVME-LC-MS/MS, Supramolecular solvent-based vortex-mixed microextraction coupled with liquid chromatography tandem mass spectrometer; ATPS-Online heart-cutting 2D-LC, aqueous two-phase system coupled with online heart-cutting two-dimensional liquid chromatography; SBSE- HPLC-DAD, stir bar sorption extraction combined with high-performance liquid chromatography-diode array detector; VA-DLLME, Vaporization assisted dispersive liquid-liquid microextraction coupled to gas chromatography-flame ionization detection; CD-DLLME-HPLC-DAD, cyclodextrin-based dispersive liquid–liquid microextraction coupled to high-performance liquid chromatography-diode array detector.

## Data Availability

The data presented in this study are available on request from the corresponding author.

## Sample Availability

Samples of the compounds are not available from the authors.
